# Limitations in next-generation sequencing-based genotyping of breast cancer polygenic risk score loci

**DOI:** 10.1038/s41431-024-01647-2

**Published:** 2024-06-21

**Authors:** Alexandra Baumann, Christian Ruckert, Christoph Meier, Tim Hutschenreiter, Robert Remy, Benedikt Schnur, Marvin Döbel, Rudel Christian Nkouamedjo Fankep, Dariush Skowronek, Oliver Kutz, Norbert Arnold, Anna-Lena Katzke, Michael Forster, Anna-Lena Kobiela, Katharina Thiedig, Andreas Zimmer, Julia Ritter, Bernhard H. F. Weber, Ellen Honisch, Karl Hackmann, Bioinformatics Working Group of the German Consortium for Hereditary Breast & Ovarian Cancer, Stephan Drukewitz, Christoph Engel, Peter Frommolt, Eva Groß, Johannes Helmuth, Zarah Kowalzyk, Maximilian Radtke, Juliane Ramser, Steffen Uebe, Shan Wang-Gohrke, Gunnar Schmidt, Marc Sturm, Corinna Ernst

**Affiliations:** 1 Institute for Clinical Genetics, University Hospital Carl Gustav Carus at TUD Dresden University of Technology and Faculty of Medicine of TUD Dresden University of Technology, Dresden, Germany; 2 ERN GENTURIS, Hereditary Cancer Syndrome Center Dresden, Dresden, Germany; 3 National Center for Tumor Diseases (NCT), NCT/UCC Dresden, a partnership between German Cancer Research Center (DKFZ), Faculty of Medicine and University Hospital Carl Gustav Carus, TUD Dresden University of Technology and Helmholtz-Zentrum Dresden-Rossendorf (HZDR), Dresden, Germany; 4 German Cancer Consortium (DKTK), Dresden, Germany; 5 German Cancer Research Center (DKFZ), Heidelberg, Germany; 6 Max Planck Institute of Molecular Cell Biology and Genetics, Dresden, Germany; 7 Department of Medical Genetics, University Hospital Münster, Münster, Germany; 8 Institute of Human Genetics, University of Regensburg, Regensburg, Germany; 9 Center for Familial Breast and Ovarian Cancer, Center for Integrated Oncology (CIO), Medical Faculty, University of Cologne and University Hospital Cologne, Cologne, Germany; 10 Department of Human Genetics, Hannover Medical School (MHH), Hannover, Germany; 11 Institute of Medical Genetics and Applied Genomics, University Hospital Tübingen, Tübingen, Germany; 12 Department of Human Genetics, University Medicine Greifswald and Interfaculty Institute of Genetics and Functional Genomics, University of Greifswald, Greifswald, Germany; 13 Department of Gynecology and Obstetrics, University Hospital Carl Gustav Carus at TUD Dresden University of Technology and Faculty of Medicine of TUD Dresden University of Technology, Dresden, Germany; 14 Department of Gynecology and Obstetrics, Institute of Clinical Chemistry Institute of Clinical Molecular Biology, University Hospital Schleswig-Holstein, Campus Kiel, Kiel, Germany; 15 Division of Gynaecology and Obstetrics, Klinikum rechts der Isar der Technischen Universität München, München, Germany; 16 Institute for Human Genetics, Medical Center University of Freiburg, Faculty of Medicine, University of Freiburg, Freiburg, Germany; 17 Department of Human Genetics, Labor Berlin – Charité Vivantes GmbH, Berlin, Germany; 18 Institute of Clinical Human Genetics, University Hospital Regensburg, Regensburg, Germany; 19 Department of Gynaecology and Obstetrics, University Hospital Düsseldorf, Heinrich-Heine University Düsseldorf, Düsseldorf, Germany; 20 Institute of Human Genetics, University of Leipzig Medical Center, Leipzig, Germany; 21 Institute for Medical Informatics, Statistics and Epidemiology, University of Leipzig, Leipzig, Germany; 22 Institute for Human Genetics, University Hospital Hamburg-Eppendorf, Hamburg, Germany; 23 Department of Obstetrics and Gynecology, Ludwig-Maximilians-University of Munich, Munich, Germany; 24 Institute of Human Genetics, Universitätsklinikum Erlangen, Friedrich-Alexander-Universität Erlangen-Nürnberg, Erlangen, Germany; 25 Department of Gynaecology and Obstetrics, University Hospital Ulm, Ulm, Germany

**Keywords:** High-throughput screening, Breast cancer, Predictive markers

## Abstract

Considering polygenic risk scores (PRSs) in individual risk prediction is increasingly implemented in genetic testing for hereditary breast cancer (BC) based on next-generation sequencing (NGS). To calculate individual BC risks, the Breast and Ovarian Analysis of Disease Incidence and Carrier Estimation Algorithm (BOADICEA) with the inclusion of the BCAC 313 or the BRIDGES 306 BC PRS is commonly used. The PRS calculation depends on accurately reproducing the variant allele frequencies (AFs) and, consequently, the distribution of PRS values anticipated by the algorithm. Here, the 324 loci of the BCAC 313 and the BRIDGES 306 BC PRS were examined in population-specific database gnomAD and in real-world data sets of five centers of the German Consortium for Hereditary Breast and Ovarian Cancer (GC-HBOC), to determine whether these expected AFs can be reproduced by NGS-based genotyping. Four PRS loci were non-existent in gnomAD v3.1.2 non-Finnish Europeans, further 24 loci showed noticeably deviating AFs. In real-world data, between 11 and 23 loci were reported with noticeably deviating AFs, and were shown to have effects on final risk prediction. Deviations depended on the sequencing approach, variant caller and calling mode (forced versus unforced) employed. Therefore, this study demonstrates the necessity to apply quality assurance not only in terms of sequencing coverage but also observed AFs in a sufficiently large cohort, when implementing PRSs in a routine diagnostic setting. Furthermore, future PRS design should be guided by the technical reproducibility of expected AFs across commonly used genotyping methods, especially NGS, in addition to the observed effect sizes.

## Introduction

The German Consortium for Hereditary Breast and Ovarian Cancer (GC-HBOC) is a consortium of interdisciplinary university centers specialized in providing counseling, genetic testing, and healthcare for individuals at risk for familial breast and ovarian cancer (BC/OC). Clinical management of women found to be at increased risk for BC/OC, due to inherited pathogenic variants in established BC/OC risk genes or a strong family history of cancer, demands for accurate and age-dependent risk estimates. Numerous studies demonstrated that the effects of BC susceptibility loci, i.e., common single nucleotide variants (SNVs) and short indels, which individually contribute only slightly to individual BC risks, but whose effects can be summed up to polygenic risk scores (PRSs), can achieve a clinically relevant degree of BC risk discrimination [1–3]. As the contribution of the PRS to BC risks has also been confirmed for carriers of a pathogenic variant in moderate- to high-penetrant BC risk genes [4–7], the inclusion of PRSs in individual BC risk prediction is increasingly implemented in GC-HBOC centers [8].

The Breast and Ovarian Analysis of Disease Incidence and Carrier Estimation Algorithm (BOADICEA), which is implemented in the CE-marked CanRisk web interface, provides (since v5) the straightforward inclusion of germline genetic test results, cancer family history, non-genetic risk factors and (if available) PRSs in a comprehensive model [9–11]. It is, therefore, widely applied for individual BC risk prediction in routine diagnostics of the GC-HBOC centers. The CanRisk web interface allows the specification of individual PRSs either as manual input (including specification of the square root of the proportion of the overall polygenic variance explained) or, for a given set of PRSs, via upload of a VCF file with the genotype or dosage information per locus to consider. Whichever method is chosen, genotyping is the responsibility of the user. For PRSs for which VCF upload is supported, CanRisk provides specifications for incorporated loci, each including the variant (chromosome, genomic position for hg19, reference and effect allele), log odds ratio (i.e., effect size), and expected AF [12]. The given alleles and AFs arise from high-throughput genotyping using one of two arrays, iCOGS13 or OncoArray [2]. The AFs are not directly included in the calculation of an individual, raw PRS, which is defined as the sum over the product of the number of observed effect alleles and corresponding effect size per PRS locus. However, observing AFs similar to the expected AFs in a sufficiently large cohort can be considered a quality criterion for PRS genotyping. The expected AFs are one of the core assumptions of the algorithm, as they determine the distribution of raw PRS values.

In the GC-HBOC centers, the BCAC 313 BC PRS, and its modified version, the BRIDGES 306 BC PRS [13], are the preferred PRS variant sets used for BC risk prediction. The germline genetic testing and genotyping of PRS loci are based on next-generation sequencing (NGS), e.g., using the TruRisk® or further specifically adapted multi-gene panels, whole-exome or whole-genome sequencing (WGS). The BRIDGES 306 BC PRS excludes loci of the original BCAC 313 BC PRS that were found not appropriately designable using NGS, some of which were replaced by corresponding loci in linkage disequilibrium [13]. The assessment of designability was mainly based on sufficient read coverage for diagnostic purposes when using a multi-gene panel approach and mapping to human reference hg19. With the implementation of BC PRS analysis in routine diagnostics and the establishment of corresponding bioinformatic workflows, further technical challenges besides insufficient coverage were identified, e.g., missing variant calls or variant calling resulting in deviating alleles. Studies systematically assessing and comparing the quality and pitfalls of germline genotyping using either arrays or NGS approaches are rare and mainly date from the early days of the establishment of NGS in clinical diagnostics [14–17]. Hence, it cannot be excluded that the conclusions drawn (which were also contradictory with regard to NGS or array being the more reliable and preferable approach) were based on now predominantly outdated technologies. Nevertheless, it is well-known that the accuracy of NGS tends to be hampered in genomic regions of low complexity, i.e., homopolymer runs, tandem repeats and strongly biased GC contents, among others [18–20]. In the Genome Aggregation Database (gnomAD), the largest and most widely used population-specific variant database, variants located in so-called low-complexity regions are flagged, to indicate that reported AFs may be erroneous [21, 22].

In this study, the Bioinformatics Working Group of the GC-HBOC conducted a systematic evaluation across GC-HBOC centers to develop a detailed, locus-wise assessment of technical pitfalls and possible sources of error in NGS-based PRS genotyping. A three-stage approach was followed. First, the AF of PRS variants was compared to the gnomAD AF for the European general population and it was checked if the variants can be converted to the hg38 reference genome. Second, PRS variant AFs in real-world data sets provided by participating GC-HBOC centers were compared to the AFs expected by CanRisk. Third, possible workarounds for use in clinical diagnostics, i.e., usage of alternative alleles and proxies, were identified. The presented results are of relevance beyond diagnostics for BC risk prediction, as they demonstrate principle difficulties in NGS-based PRS computation, especially for PRSs developed based on array data. Furthermore, the results underline the necessity of a comprehensive technical evaluation of PRS variant genotyping in clinical use, as the predictive ability of an individual PRS crucially depends on the assumptions made about the underlying AFs.

## Materials and methods

### Variant annotation

For denoting variants, dbSNP identifiers and gnomAD-like annotations were used throughout the manuscript. The corresponding HGVS annotations are listed in Supplementary Table 1.

### Evaluation of expected allele frequencies & convertibility to hg38

Two BC PRS variant sets were considered, namely, the BCAC 313 and the BRIDGES 306 BC PRS. Of the two sets, 295 loci are identical, 18 loci are unique to BCAC 313 BC PRS, and further 11 loci are unique to the BRIDGES 306 BC PRS, resulting in a total number of *N* = 324 variants to be considered. Expected AFs were extracted from the corresponding PRS specification files at the CanRisk knowledge base [12]. Additionally, AFs in the non-Finnish European (NFE) general population were obtained from the gnomAD v3.1.2 database^1^, which are based on more than 33,000 WGS samples mapped to the hg38 reference sequence. For conversion of the hg19-based PRS variants from CanRisk to hg38, the gnomAD liftover feature was used.

Besides AFs, gnomAD flags and warnings indicating possible technical artifacts were retrieved and recorded. These included localization within low-complexity regions, low-quality sites (i.e., sites that are covered in <50% of considered samples [21]), and sites not passing the allele-specific GATK Variant Quality Score Recalibration (VQSR) filter.

### Determination of deviating allele frequencies

To determine PRS variants with considerably deviating AFs, thresholds had to be defined dependent on sample sizes and variances observed. Therefore, individual thresholds per data set were determined, using an elbow of the curve method. The absolute differences between observed and expected AFs were sorted in descending order, and the absolute difference referring to the point with the largest Euclidean distance to the imaginary line between thought points (0, 1) and (N + 1, 0) was chosen as threshold, i.e., all observed absolute differences greater than this threshold were determined as noticeably deviating. Corresponding curves are shown in Supplementary Figs. 1–6. If the same set of samples was processed with two different variant callers, the smaller threshold was applied in each case, to facilitate comparing variant caller performance.

### Real-world data collection

Genotyping results for either BCAC 313 or BRIDGES 306 BC PRS loci in a cohort of at least 100 individuals of European ancestry were requested from GC-HBOC centers. Family IDs were checked for uniqueness to prevent samples from related individuals. Participating centers submitted observed AFs per locus as well as fractions of samples that did not meet the required quality criteria (e.g., with regard to minimum read depth). Furthermore, details on sequencing approaches and bioinformatic analysis workflows for PRS genotyping were systematically recorded.

In total, five GC-HBOC centers provided data, namely the Institute of Medical Genetics and Applied Genomics (IMGAG), University Hospital Tübingen, the Institute for Clinical Genetics (ICG), University Hospital Carl Gustav Carus Dresden, the Department of Medical Genetics (DMG) at University Hospital Münster, the Center for Familial Breast and Ovarian Cancer (CFBOC), University Hospital Cologne, and the Institute of Human Genetics (IHG) at the University of Regensburg. Each center provided two NGS-based data sets. An overview of data characteristics is given in Table 1. A more detailed description of sample compositions, sequencing approaches and bioinformatic analyses can be found in Supplementary Methods.

**Table 1:** Characteristics of data sets provided by participating centers of the German Consortium for Hereditary Breast & Ovarian Cancer (GC-HBOC), namely the Institute of Medical Genetics and Applied Genomics (IMGAG), University Hospital Tübingen, the Institute for Clinical Genetics (ICG), University Hospital Carl Gustav Carus Dresden, the Department of Medical Genetics (DMG), University Hospital Münster, the Center for Familial Breast and Ovarian Cancer (CFBOC), University Hospital Cologne, and the Institute of Human Genetics (IHG) at the University of Regensburg.

		IMGAG	ICG	DMG	CFBOC	IHG
Sample size	Set 1	348	585	545	412	251
	Set 2	1410				
Testing indication		Various	Cancer-related	Familial BC/OC	Familial BC/OC	Familial BC/OC
Considered PRS		BCAC 313	BCAC 313	BRIDGES 306	BRIDGES 306	BRIDGES 306
			BRIDGES 306			
NGS approach		WGS	Twist Custom Cancer Panel	Twist Custom Panel	Agilent TruRisk v3	Agilent TruRisk v3
Reference		hg38	hg19	hg19	hg19	hg38
Variant caller	Set 1	DRAGEN v4.0.3	freebayes v1.3.6	DRAGEN v4.2.4	freebayes v1.3.6	CLC LightSpeed v23.0.2
	Set 2	freebayes v1.3.6	GATK v4.2.6 HaplotypeCaller	GATK v4.4.0 HaplotypeCaller	GATK v4.3.2 HaplotypeCaller	GATK v4.2.6 HaplotypeCaller
Calling mode	Set 1	Unforced	Forced	Forced	Forced	Unforced
	Set 2					Forced
Quality filter		DP ≥ 15	DP ≥ 20	DP ≥ 20	DP ≥ 30	DP ≥ 10

Each center provided two data sets.

*BC*/*OC* breast/ovarian cancer, *DP* sequencing depth, *PRS* polygenic risk score.

### Assessment of effects of deviating allele frequencies on estimated breast cancer risks

Effects of noticeably deviating AFs of PRS loci on CanRisk-based estimated BC risks rely on the number and combination of affected loci, as well as a multitude of additional risk factors such as results of germline testing of established BC/OC risk genes, BC/OC family history, non-genetic risk factors, and current age. Principally, the proportional contribution of the PRS to overall BC risk decreases with increasing age, and also decreases for carriers of a germline pathogenic variant in a BC risk gene with moderate to high penetrance [10]. In order to get an estimate of expected biases in predicted BC risks due to potentially erroneous PRS genotyping, estimates of 10-year and remaining lifetime risks, i.e., cumulative risks of primary BC until age of 80 years, were calculated for imaginary, cancer-unaffected women of three different ages, namely 20, 40, and 60 years, without any further information than (artificial) PRS.

To simulate different scenarios, artificial VCF files were constructed with an average PRS (50th percentile) by setting dosage to two times the expected CanRisk AF using the DS tag. For each data set, for loci showing noticeably deviating AFs, DS was set to two times the observed AF in the data set. Dates of birth were set to January 1 in 2004, 1984, and 1964, to simulate 20, 40, and 60 years of age at the time of risk computation, which were performed in March 2024, using the web interface of CanRisk v2.3.5, and under specification of the default UK incidence rates.

### Elaboration of workarounds

Potential solutions for improving genotyping performance with respect to expected AFs could be (besides improving the calling itself) the consideration of alternative alleles or proxies. Details on the identification of potential variants to substitute for this purpose are given in Supplementary Methods. Alternative variants in gnomAD v.3.1.2 with an AF matching the expected CanRisk AF were further evaluated using the IMGAG freebayes data, as this (i) was the largest data set in the study (*n* = 1410), and (ii) the only WGS-based data set, which allowed genotyping of the entire set of putative proxies.

## Results

### Missing loci & convertibility to hg38

For four BC PRS loci, no variants were listed at the specified genomic position in gnomAD v2.1.1, namely rs572022984, rs113778879, rs73754909, and rs79461387. gnomAD v3.1.2 also reported no variants for three of these four loci for corresponding loci in hg38 as defined by dbSNP [23] (Supplementary Table 2). Locus rs572022984 was listed but with an overall allele count of zero in NFE samples (Table 2).

**Table 2:** Characteristics of loci incorporated in the BCAC 313 or BRIDGES 306 breast cancer PRSs that were either not included in the gnomAD v3.1.2 database or reported with extremely deviating allele frequency compared to CanRisk.

		CanRisk log OR			gnomAD v3.1.2	
rs ID	Locus (hg19)	BCAC	BRIDGDES	AF	AF	Comment
rs56168262	1-51467096-CT-C	0.0374	0.0374	0.4856	0.3969	LCR
rs56097627	1-110198129-CAAA-C	0.0458	0.0458	0.7779	0.0681	LCR
rs143384623	1-145604302-C-CT	−0.0399	−0.0399	0.3490	0.3764	LCR
rs78425380	2-10138983-T-C	0.0603		0.1168	0.0085	LCR, LQS
rs553796823	2-39699510-C-CT	−0.0402	−0.0402	0.4647	0.5134	LCR
rs572022984	2-217955896-GA-G	−0.2016	−0.2016	0.0364	Allele count zero	
rs774021038	4-84370124-TA-T	−0.0464	−0.0464	0.5353	0.5030	LCR
rs147404208	4-92594859-TTCTTTC-T	−0.0407		0.4386	0.4911	LCR
rs62331150	4-106069013-G-T	0.0471	0.0471	0.2286	0.4214	LQS
rs113778879	5-58241712-C-T	−0.0434		0.5762	Not listed in gnomAD	
rs543824204	6-20537845-CA-C	−0.0391	−0.0391	0.4741	0.3405	LCR
rs574103382	6-82263549-AAT-A	0.0477		0.4240	0.3242	LCR
rs73754909	6-87803819-T-C	0.0383	0.0383	0.2809	Not listed in gnomAD	
rs60954078	6-151955914-A-G	0.1449	0.1449	0.0726	0.1519	LCR
rs57589542	6-152022664-CAAAAAAA-C	0.0137	0.0137	0.6130	0.5048	LCR
rs10644978	7-91459189-A-ATT	0.0452	0.0452	0.3332	0.3675	LCR
rs111963714	7-99948655-T-G	0.0420	0.0420	0.2083	0.1425	
rs5887960	7-139943702-CT-C	0.0582	0.0582	0.5378	0.4091	LCR
rs3988353	8-17787610-CT-C	−0.0377	−0.0377	0.6217	0.4462	LCR, VQSR
rs3057314	9-21964882-CAAAA-C	0.0550	0.0550	0.3210	0.2794	LCR
rs2384736	10-38523626-C-A	0.0404	0.0404	0.3740	0.0003	LCR, LQS
rs111833376	10-71335574-C-T	−0.0404	0.3122	0.3122	0.0699	LCR
rs140936696	10-95292187-CAA-C	−0.0512	−0.0512	0.8177	0.7074	LCR
rs10862899	12-85004551-C-T	0.0348	0.0348	0.4999	0.5259	
rs57920543	16-4008542-CAAAAA-C	−0.0329	−0.0329	0.8194	0.7400	LCR
rs79461387	17-29168077-G-T	−0.0568	−0.0568	0.2573	Not listed in gnomAD	
rs2668667	17-44283858-G-A	−0.0540	−0.0540	0.1919	0.1586	
rs112855987	22-45319953-G-A	−0.0134		0.4158	0.5272	LCR

Log odds ratios (ORs) are identical for BCAC 313 and BRIDGES 306, but missing values indicate loci not included in the corresponding PRS. Entries in the Comment column refer to technical artifacts reported in gnomAD.

*LCR* low-complexity region, *LQS* low-quality site (in <50% of samples covered), *VQSR* failed allele-specific GATK Variant Quality Score Recalibration (VQSR) filter.

For two loci, conversion to hg38 resulted in a change in alleles, namely for rs143384623 (hg19: 1-145604302-C-CT; hg38: 1-145830798-C-CA) and rs550057 (hg19: 9-136146597-C-T; hg38: 9-133271182-T-C). For rs143384623, the change of the alternative allele from CT to CA did not result in a noticeable shift in AFs observed in gnomAD NFE samples (5142/13304 (0.39) in v2.1.1 versus 24316/64610 (0.38) in v3.1.2, two-sided Fisher’s exact test *p* = 0.14). For rs550057, the observed AFs appeared exactly opposite, i.e., 3786/14828 (0.26) for allele T in gnomAD v2.1.1 and 49878/67552 (0.74) for allele C in gnomAD v3.1.2. Therefore, 1 − 49878/67552 was assumed as the gnomAD v3.1.2 effect AF at this bi-allelic site.

### Allele frequencies & technical artifacts reported in gnomAD v3.1.2

For 39 of the 320 PRS loci listed with AF > 0 in gnomAD v3.1.2, at least one observation of technical artifacts was reported: 38 loci were flagged as being located in low-complexity regions, 3 as being localized at a low-quality site, and 1 failed the allele-specific VQSR filter (Supplementary Table 2).

**Fig. 1:**
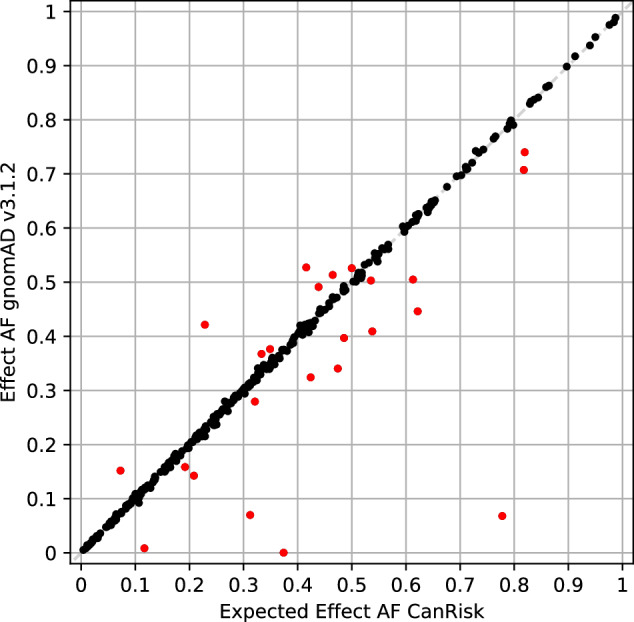
Comparison of variant effect allele frequencies (AFs) specified by CanRisk and observed in gnomAD v3.1.2 non-Finnish European samples for 320 variants incorporated in BCAC 313 or BRIDGES 306 breast cancer polygenic risk scores. Extremely deviating AFs with an absolute difference > 0.016 are indicated by red markers.

Due to the absolute difference threshold 0.016 (Supplementary Fig. 1), 24 loci were determined as showing deviating AFs compared to CanRisk (Fig. 1, Table 2). Absolute differences ranged from 0.03 to 0.71, and for 21 out of these 24 loci (87.5%), technical artifacts were reported in gnomAD v3.1.2.

### Evaluation of real-world next-generation sequencing outcome

All 49 PRS loci for which a noticeably deviating AF was observed in at least one of the data sets provided by the five participating GC-HBOC centers are listed in Table 3.

**Table 3:** Summary of polygenic risk score genotyping results with noticeably deviating allele frequencies (AFs) of centers of the German Consortium for Hereditary Breast and Ovarian Cancer.

rs ID	Locus (hg19)			Allele frequencies											gnomAD
		log OR			IMGAG (WGS)		ICG (MGP)		DMG (MGP)		CFBOC (MGP)		IHG (MGP)		deviating
		BCAC	BRIDGES	Expected	DRAGEN	FB	GATK	FB	GATK	DRAGEN	GATK	FB	GATK	CLC	AF
rs56168262	1-51467096-CT-C	0.0374	0.0374	0.4856	0.4468	**0.4025**	0.4653	0.4846	0.4907	**0.1193**	**0.3496**	0.4922	0.4940	**0.2211**	Yes
rs56097627	1-110198129-CAAA-C	0.0458	0.0458	0.7779	**0.0560**	**0.0599**	–	**0.4299**	**0.1692**	**0.0284**	**0.2368**	**0.3898**	**0.4880**	**0.1992**	Yes
rs12406858	1-118141492-A-C	0.0452	0.0452	0.2654	0.2874	**0.3096**	0.2846	0.2846	0.2523	0.2523	0.2658	0.2670	0.2550	0.2948	No
rs143384623	1-145604302-C-CT	−0.0399	−0.0399	0.3490	0.3578	0.3628	–	0.3904	**0.4941**	0.3651	0.3467	**0.3010**	–	–	Yes
rs11463354	1-172328767-T-TA	−0.0435	−0.0435	0.3264	0.3305	0.3142	**0.2637**	0.3880	**0.4496**	0.3211	**0.2694**	**0.2427**	0.3127	0.3167	No
rs11268668	see caption	−0.0321	−0.0321	0.7983	0.8233	**0.5433**	0.8009	**0.4803**	0.7917	0.7917	0.8070	**0.4769**	0.7908	0.7709	No
rs553796823	2-39699510-C-CT	−0.0420	−0.0420	0.4647	**0.5603**	0.4865	0.5000	**0.3641**	0.4956	**0.0972**	**0.3710**	**0.3856**	0.4602	**0.3247**	Yes
rs11693806	2-218292158-C-G	−0.0757	−0.0757	0.7289	0.7457	0.7443	0.7675	0.7675	0.7358	0.7358	0.7112	0.7112	**0.4980**	–	No
rs371314787	3-49709912-C-CT	−0.0367	−0.0367	0.2847	0.2917	0.2809	0.2305	0.2274	**0.4889**	0.2376	0.2476	**0.2354**	**0.4980**	0.2490	No
rs34207738	3-141112859-CTT-C	0.0551	0.0551	0.4205	0.3980	0.3929	0.4265	**0.3316**	0.4566	0.4248	0.4238	0.3800	0.3805	0.3745	No
rs774021038	4-84370124-TA-T	−0.0464	−0.0464	0.5353	0.4871	**0.4840**	0.5420	0.5214	**0.0000**	–	**0.4757**	**0.4539**	0.4741	0.4741	Yes
rs147399132	4-126752992-A-AAT	−0.0377		0.5123	0.4842	0.5092	0.4909	**0.3880**							No
rs199562199	5-52679539-C-CA	0.0571	0.0571	0.1001	0.1049	0.1142	0.1527	**0.4376**	**0.1829**	0.0642	0.1262	**0.1711**	**0.3924**	0.1195	No
rs113803968	5-55662540-C-CT	−0.0458	−0.0458	0.3657	0.4066	0.3603	**0.3018**	**0.2957**	**0.4919**	0.3422	0.3350	**0.2864**	**0.2948**	0.3028	No
rs113778879	5-58241712-C-T	−0.0434		0.5762	**0.0000**	**0.0000**	**0.0000**	**0.0015**							–
rs10074269	5-169591460-T-C	0.0412	0.0412	0.3393	0.3463	0.3507	0.3538	0.3538	0.3349	0.3349	0.3398	0.3398	**0.2709**	**0.2709**	No
rs543824204	6-20537845-CA-C	−0.0391	−0.0391	0.4741	**0.2802**	**0.2904**	0.4412	0.5000	0.5027	**0.3119**	**0.4022**	0.4951	0.5000	**0.2649**	Yes
rs574103382	6-82263549-AAT-A	0.0477		0.4240	**0.3391**	**0.3564**	**0.3193**	0.3880							Yes
rs73754909	6-87803819-T-C	0.0383	0.0383	0.2809	**0.0000**	**0.0004**	**0.0000**	**0.0148**	**0.0734**	0.2615	**0.0000**	**0.0350**	**0.0020**	–	–
rs55941023	6-130341728-C-CT	0.0472	0.0472	0.7113	0.7414	0.7099	0.7154	0.7154	0.6899	0.6899	0.7197	0.7197	**0.7809**	**0.7809**	No
rs57589542	see caption	0.0137	0.0137	0.6130	**0.3908**	**0.3723**	**0.3488**	**0.3632**	**0.3940**	**0.2954**	**0.3400**	**0.3204**	**0.3506**	**0.2928**	Yes
rs10644978	7-91459189-A-ATT	0.0452	0.0452	0.3332	0.3520	0.3585	0.3134	0.3274	**0.4623**	0.3514	0.3325	0.3313	0.3566	0.3685	Yes
rs111963714	7-99948655-T-G	0.0420	0.0420	0.2083	**0.1494**	0.2113	–	–	**0.1002**	**0.1523**	**0.1493**	0.2027	0.1793	**0.0339**	Yes
rs5887960	7-139943702-CT-C	0.0582	0.0582	0.5378	**0.4468**	**0.4330**	**0.4700**	0.4752	**0.4804**	**0.3890**	0.5085	**0.4757**	0.4801	**0.4084**	Yes
rs62485509	7-144048902-G-T	−0.0563	−0.0563	0.2289	**0.1595**	0.2255	0.2265	0.2256	0.2229	**0.1092**	0.2354	0.2354	0.2490	0.1753	No
rs3988353	8-17787610-CT-C	−0.0377	−0.0377	0.6217	**0.5460**	**0.5479**	**0.4345**	**0.4214**	**0.5089**	**0.2495**	**0.3568**	**0.4187**	**0.4960**	**0.1534**	Yes
rs1511243	8-76230943-A-G	0.0755	0.0755	0.8289	0.8348	0.8376	0.8333	0.8333	0.8376	0.8376	0.8374	0.8374	**0.6175**	0.8486	No
rs10975870	9-6880263-A-G	0.0348	0.0348	0.2900	0.3132	0.2848	0.2975	0.2975	0.3018	0.3018	0.2532	0.2524	0.2968	**0.1972**	No
rs3057314	9-21964882-CAAAA-C	0.0550	0.0550	0.3210	**0.2011**	**0.2043**	**0.1803**	**0.1923**	0.3105	**0.1101**	**0.1667**	**0.0595**	**0.4183**	**0.0896**	Yes
rs4880038	9-36928288-T-C	0.0249	0.0249	0.5427	0.5431	0.5440	0.5026	0.5026	0.5165	0.5165	0.5388	0.5376	**0.4024**	0.5080	No
rs542275778	10-22477776-ACC-A	0.1687	0.1687	0.0214	0.0187	0.0294	0.0318	0.0256	0.0435	0.0294	**0.1535**	0.0317	**0.1474**	0.0299	No
rs2384736	10-38523626-C-A	0.0404	0.0404	0.3740	**0.0014**	0.3996	**0.0009**	0.3521	**0.0000**	**0.1817**	**0.0000**	**0.0836**	**0.0020**	**0.0020**	Yes
rs111833376	10-71335574-C-T	−0.4040	−0.4040	0.3122	**0.0417**	**0.0443**	0.2681	0.2684	**0.2561**	**0.1128**	**0.0181**	0.3051	**0.0558**	**0.2231**	Yes
rs140936696	10-95292187-CAA-C	−0.0512	−0.0512	0.8177	0.7677	**0.7742**	–	–	**0.4492**	**0.1853**	0.8232	**0.4746**	**0.4821**	**0.2590**	Yes
rs9421410	10-123095209-G-A	−0.0538	−0.0538	0.3246	0.3247	0.3170	0.3068	0.3068	0.2761	0.2752	0.3058	0.3058	**0.2590**	–	No
rs35054928	10-123340431-GC-G	−0.2408	−0.2408	0.5971	0.5747	0.6028	0.5744	0.5744	0.5477	0.5477	**0.5206**	**0.5206**	0.5418	0.5438	No
rs199504893	11-108267402-C-CA	−0.0022		0.4168	0.4526	0.4362	0.3877	**0.3137**							No
rs11049431	12-28347382-C-T	−0.0521	−0.0521	0.2151	0.1997	0.2053	–	–	0.2138	0.2128	0.1735	0.1735	0.1693	**0.1175**	No
rs1027113	12-29140260-G-A	0.0647	0.0647	0.9124	0.9109	0.9195	0.9162	0.9162	0.9284	0.9284	0.9163	0.9163	**0.7649**	0.9024	No
rs144767203	15-100905819-A-C	−0.0608	−0.0608	0.1072	0.0934	0.1043	–	–	0.1112	0.1046	0.1363	0.1371	0.0837	**0.0199**	No
rs57920543	16-4008542-CAAAAA-C	0.0550	0.0550	0.8194	**0.7457**	**0.7011**	**0.4668**	**0.4658**	**0.4758**	**0.4064**	0.7959	**0.4830**	**0.4761**	**0.4442**	Yes
rs12709163	16-6963972-C-G	0.0354	0.0354	0.7915	0.7572	0.7660	0.7846	0.7846	0.7982	0.7982	0.7791	0.7791	**0.6932**	0.7988	No
rs9931038	16-85145977-T-C	−0.0211	−0.0211	0.4851	**0.5431**	0.5110	0.4855	0.4855	0.4734	0.4734	0.4757	0.4757	0.4940	0.4940	No
rs79461387	17-29168077-G-T	−0.0568	−0.0568	0.2573	**0.0000**	0.2567	0.2504	0.2504	**0.0257**	0.2385	**0.0000**	0.2494	–	–	–
rs71363517	17-43212339-C-CT	0.0438	0.0438	0.2273	0.2256	0.2128	–	–	**0.4975**	0.2101	0.2017	0.2106	**0.2928**	0.2012	No
rs2668667	17-44283858-G-A	−0.0540	−0.0540	0.1919	**0.0805**	0.1872	–	–	0.1963	0.1413	0.1808	0.1808	0.1813	**0.0876**	Yes
rs1111207	18-24125857-T-C	0.0346	0.0346	0.4243	0.4267	0.4135	0.4282	0.4282	0.4321	0.4321	0.4345	0.4345	**0.3267**	0.4104	No
rs140702307	19-19517054-C-CGGGCG	0.0437	0.0437	0.3525	0.3405	0.3504	0.3735	0.3000	0.3450	0.3468	0.3410	**0.2694**	0.3386	0.3247	No
rs66987842	22-40904707-CT-C	0.1148	0.1148	0.1068	0.1207	0.1195	0.1160	0.1188	**0.1820**	0.1404	0.1141	0.1165	0.1414	0.1016	No

Noticeably deviating AFs are shown in bold. Loci (hg19-based) of rs11268668 and rs57589542 are 1-204502514-T-TTCTGAAACAGGG (hg19) and 6-152022664-CAAAAAAA-C (hg19), respectively.

*WGS* whole-genome sequencing, *MGP* multi-gene panel sequencing, *FB* freebayes.

**Fig. 2:**
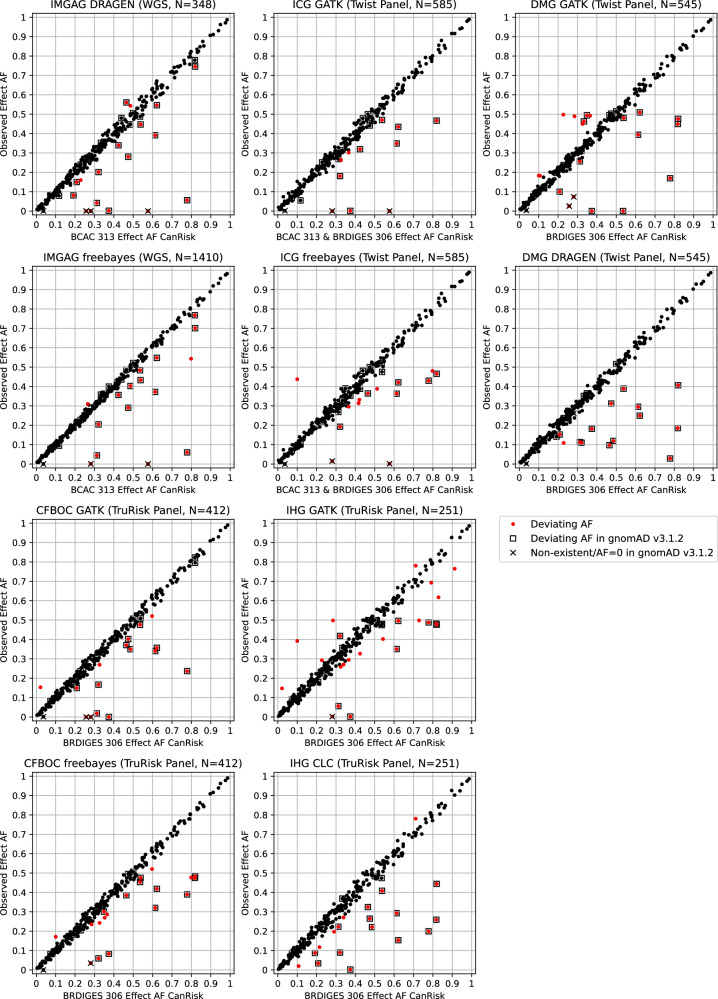
Comparison of effect allele frequencies (AFs) specified by CanRisk and observed in ten real-world data sets for 320 loci incorporated in BCAC 313 or BRIDGES 306 breast cancer polygenic risk scores. Data were provided by the Institute of Medical Genetics and Applied Genomics (IMGAG) at University Hospital Tübingen, Institute for Clinical Genetics (ICG) at University Hospital Carl Gustav Carus Dresden, by the Department of Medical Genetics (DMG) at University Hospital Münster, by the Center for Familial Breast and Ovarian Cancer (CFBOC) at University Hospital Cologne, and by the Institute of Human Genetics (IHG) at the University of Regensburg.

For the IMGAG DRAGEN data, 0.052 was calculated as threshold to determine noticeably deviating AFs (Supplementary Fig. 2), resulting in 18 loci affected (Table 3, Fig. 2). Of these, 16 were previously also identified as missing or showing noticeably deviating AFs in gnomAD v3.1.2. The exceptions were rs62485509 and rs9931038. For IMGAG freebayes data, 0.036 was calculated as threshold (Supplementary Fig. 2), resulting in 16 loci from the BCAC 313 BC PRS determined as showing a noticeably deviating AF. Of these, 11 loci were also identified as showing deviating AF in IMGAG DRAGEN data, and all but rs12406858 and rs11268668 were previously identified as missing or showing deviating AFs in gnomAD v3.1.2.

Considering genotyping data provided by the ICG based on 585 samples, 23 of the overall 324 PRS loci did not meet the minimum quality criteria (read depth ≥ 20) in more than 25% of samples and were discarded (Supplementary Table 3). Additionally, GATK reported read depth <20 for >25% of samples for rs56097627 and rs143384623. For 260 of the remaining 299 PRS loci (86.96%), forced genotyping with GATK and freebayes resulted in the observation of identical AFs. For both ICG GATK and freebayes data, 0.063 was calculated as threshold to determine noticeably deviating AFs (Supplementary Fig. 3). Using this threshold, 11 loci showed noticeably deviating AFs in the GATK data set (including two loci exclusive for BCAC 313 BC PRS) and 14 loci in the freebayes data set (including three loci exclusive for BCAC 313 BC PRS), respectively, with an overlap of 7 (Table 3, Fig. 2).

The DMG provided GATK- and DRAGEN-based BRIDGES 306 BC PRS genotyping data of 545 samples. Locus rs138179519 did not meet the quality criteria, and additionally rs774021038 using DRAGEN. Of the remaining 304 loci, 252 (82.89%) showed identical AFs (Supplementary Table 3). Using a threshold of 0.052 (Supplementary Fig. 4), resulted in 20 loci showing deviating AFs in GATK data and 14 loci in DRAGEN data, respectively, with an overlap of 9 loci.

For the CFBOC data based on 412 samples, a threshold of 0.047 was calculated (Supplementary Fig. 5). The loci of the BRIDGES 306 BC PRS were considered, 243 (79.41%) of which showed identical AFs for both callers applied (Supplementary Table 3). Overall 25 loci (all of which are included also in the BCAC 313 BC PRS) showed deviating AFs: 16 loci in GATK and 19 loci in freebayes data, with an overlap of 10 loci.

The IHG provided GATK- and CLC-based BRIDGES 306 BC PRS genotyping data of 251 samples (Supplementary Methods). Four loci did not meet the quality criteria in both settings, and additional four in the CLC setting. Of the remaining 298 loci, 228 (76.51%) showed identical AFs (Supplementary Table 3). Using a threshold of 0.063 (Supplementary Fig. 6), resulted in 23 loci showing noticeably deviating AFs in GATK data, respectively 19 loci in CLC data, with an overlap of 10 loci.

In summary, for four loci, deviating AFs were reported in all GC-HBOC real-world settings examined, namely for rs56097627, rs113778879, rs57589542, and rs3988353. Further four loci, namely rs574103382, rs73754909, rs3057314, and rs57920543, were reported with deviating AFs in all settings except for one (Table 3).

However, there were also 16 loci that were conspicuous in a single setting exclusively, namely five in IHG GATK data (rs1511243, rs4880038, rs1027113, rs12709163, rs1111207), three each in ICG freebayes data (rs34207738, rs147399132, rs199504893) and in IHG CLC data (rs10975870, rs11049431, rs144767203), two in DMG GATK data (rs10644978, rs66987842), and one each in IMGAG DRAGEN (rs9931038), IMGAG freebayes data (rs12406858), and CFBOC freebayes data (rs140702307). Another three loci (rs10074269, rs55941023, rs35054928) showed AF deviations in only one center, but these were concordant.

Considering the loci non-existent in gnomAD v3.1.2, rs113778879 was not observed with expected AF in any GC-HBOC center, and rs73754909 only with forced DRAGEN calling in DMG data. For rs79461387, expected AFs were reported consistently when using freebayes, but not by unforced DRAGEN calling and in two settings using forced GATK. Of note, rs572022984 with zero allele count in gnomAD v3.1.2 NFEs and an expected AF of 0.0364 in CanRisk, was consistently not observed at all or with a maximum AF of 0.0037 (Supplementary Table 3).

Five loci showing aberrant AFs in gnomAD v3.1.2 NFEs (Table 2) were not reported with deviating AF by any of the participating GC-HBOC centers, namely rs78425380, rs62331150, rs60954078, rs10862899, and rs112855987.

### Implications on risk prediction

Without further information and assuming a standardized PRS at the 50th percentile, the estimated 10-year risks of developing primary BC of cancer-unaffected women of 20, 40, and 60 years of age were 0.1%, 1.5%, and 3.4% according to CanRisk (Supplementary Table 4). Percentiles of PRSs from artificial VCF files with aberrant dosages (see “Materials and Methods”) ranged from 47.5% (IHG CLC, BRIDGES 306) up to 55.7% (ICG freebayes, BCAC 313). The risk of 0.1% for a 20-year-old woman was concordantly unchanged in all scenarios including artificial PRSs. For a 40-year-old woman, estimated 10-year risks were increased by 0.1% in seven scenarios, and for a 60-year-old woman by up to 0.2% in eight scenarios.

Estimated remaining lifetime risks of developing primary BC assuming an average PRS (50th percentile) of cancer-unaffected women aged 20, 40, and 60 years are 11.3%, 10.9%, and 7.1% according to CanRisk (Supplementary Table 4). When using PRSs from artificial VCF files with aberrant dosages, estimated lifetime risks ranged from 11.1% up to 11.9% for a 20-year-old woman, from 10.6% up to 11.4% for a 40-year-old woman, and from 7.0% up to 7.4% for a 60-year-old woman. The lowest estimates were obtained with the BRIDGES 306 BC PRS based on IHG CLC data with 19 artificial dosages imputed, and the highest with the BCAC 313 BC PRS based on ICG freebayes data with 14 artificial dosages imputed.

### Consideration of alternative alleles and loci in linkage disequilibrium

**Fig. 3:**
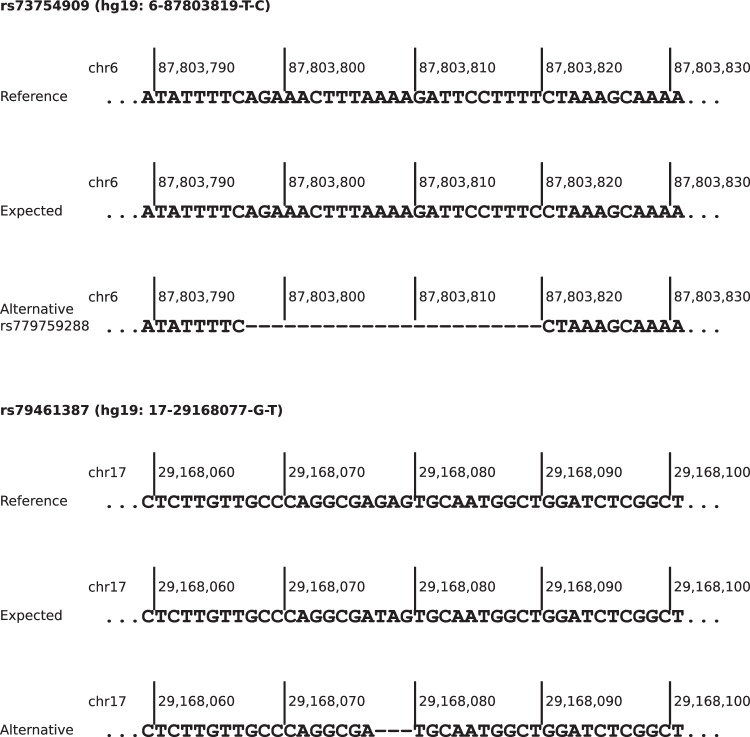
Sequences of reference, expected effect allele and potential alternative allele of polygenic risk score loci rs73754909 and rs79461387 (hg19-based). Both alternative alleles are deletions with the adjacent downstream nucleotide identical to the expected substituted one.

For 20 PRS loci showing noticeably deviating AFs in at least one real-world NGS data set, alternative alleles or overlapping variants with minimum AF 0.01 in NFEs were reported in gnomAD v3.1.2 (Supplementary Table 5). For rs73754909 and rs79461387, both SNVs and non-existent in gnomAD v3.1.2, deletions were reported with comparable AFs to the ones expected by CanRisk. For both deletions, the adjacent downstream nucleotide of the reference sequence was identical to the substituted nucleotide of the expected effect allele (Fig. 3). For rs113778879, which is also an SNV not contained in gnomAD v3.1.2, a similar observation could be made (Supplementary Fig. 7), but the reported AF exceeds the expected one by more than 0.1 (0.5762 versus 0.6818).

For 28 out of the 49 loci showing noticeable deviating AFs in at least one real-world data set, proxies in 1000G GRCh37 microarray data, 1000G GRCh38 High Coverage WGS data, or TOPMED European data could be identified (Supplementary Table 6). For rs113778879, rs73754909, and rs79461387, LDpair based on GRCh38 reported the same alternative alleles as gnomAD v3.1.2 (Supplementary Table 5), where the original PRS loci are non-existent.

Proxies and alternative alleles showing AFs in gnomAD v3.1.2 comparable to expected CanRisk AFs, i.e., an absolute deviation <0.016, were considered as possible workarounds for improved PRS genotyping, and further evaluated with respect to observed AFs in IMGAG freebayes data (Table 4). For 19 of these 21 PRS loci, absolute differences between expected and observed AFs in IMGAG freebayes data remained below the previously defined IMGAG freebayes-specific threshold of 0.036. The exceptions were the substitutions of rs12406858 and rs79461387. The latter is noteworthy because the original PRS locus, which is an SNV, was correctly called by freebayes in forced and unforced mode (Table 3), whereas GATK HaplotypeCaller seemed to call an overlapping deletion of sequence GAG in DMG and CFBOC data. Also noteworthy are the potential replacements of rs73754909 and rs111833376, as both variants were called with noticeably deviating AFs in most real-world data sets.

**Table 4:** Potential solutions for improving polygenic risk score (PRS) genotyping performance with respect to the achievement of allele frequencies (AFs) expected by CanRisk, using alternative alleles or proxies.

rs ID	Locus (hg19)	Expected AF	Workaround	gnomAD AF	IMGAG FB AF
rs12406858	1-118141492-A-C	0.2654	Proxy rs1966228	0.2622	0.3064
rs11693806	2-218292158-C-G	0.7289	Proxy rs3821098	0.7422	0.7443
rs34207738	3-141112859-CTT-C	0.4205	Summing up the AFs of deletions of two and three thymines	0.4344	0.4167
rs10074269	5-169591460-T-C	0.3393	Proxy rs4562056	0.3414	0.3511
rs73754909	6-87803819-T-C	0.2809	Alternative allele rs77846138^a^	0.2846	0.2578
			Proxy rs12664322	0.2849	0.2791
rs55941023	6-130341728-C-CT	0.7113	Proxy rs1415700	0.7049	0.7050
			Proxy rs11390217	0.7058	0.7046
rs1511243	8-76230943-A-G	0.8289	Proxy rs6472903	0.8294	0.8376
rs10975870	9-6880263-A-G	0.2900	Proxy rs12380608	0.2863	0.2840
			Proxy rs10975887	0.2849	0.2837
rs4880038	9-36928288-T-C	0.5427	Proxy rs4880039	0.5449	0.5436
			Proxy rs7032313	0.5446	0.5440
rs542275778	10-22477776-ACC-A	0.0214	Proxy rs112287594	0.0185	0.0270
rs111833376	10-71335574-C-T	0.3122	Summing up AFs of rs111833376 and rs753981427^b^	0.3200	0.3163
			Proxy rs12769661	0.2984	0.2929
rs9421410	10-123095209-G-A	0.3246	Proxy rs7913694	0.3142	0.3110
			Proxy rs35098964	0.3139	0.3099
rs35054928	10-123340431-GC-G	0.5971	Proxy rs2981579	0.5908	0.5996
rs11049431	12-28347382-C-T	0.2151	Proxy rs11049519	0.2142	0.2039
rs144767203	15-100905819-A-C	0.1072	Proxy rs58855876	0.1078	0.1043
			Proxy rs113438754	0.1078	0.1043
rs12709163	16-6963972-C-G	0.7915	Proxy rs1492386	0.7951	0.7684
rs9931038	16-85145977-T-C	0.4851	Proxy rs60296580	0.4903	0.5082
rs79461387	17-29168077-G-T	0.2573	Alternative allele rs550458309^c^	0.2719	0.0000
rs2668667	17-44283858-G-A	0.1919	Proxy rs2532237	0.1860	0.1957
			Proxy rs150290194	0.1765	0.1858
rs1111207	18-24125857-T-C	0.4243	Proxy rs1111208	0.4249	0.4135
rs66987842	22-40904707-CT-C	0.1068	Proxy rs6001949	0.1003	0.1195

Resulting AFs were investigated based on gnomAD v3.1.2 non-Finnish European data and genotyping results of 1410 European whole-genome sequencing (WGS) samples using (unforced) freebayes (FB), provided by the Institute of Medical Genetics and Applied Genomics (IMGAG) at University Hospital Tübingen.

^a^6-87094100-CAGAAACTTTAAAAGATTCCTTTT-C (hg19).

^b^10-71335572-TCC-T (hg19).

^c^17-29168076-AGAG-A (hg19).

## Discussion

This study describes the systematic evaluation of NGS-based PRS genotyping in real-world data sets of five GC-HBOC centers. The observed AFs of PRS loci in individuals with European descent were used as quality criterion, as the reproducibility of expected AFs of the PRS loci, and hence, the assumptions made about the overall PRS distribution, are an essential prerequisite for a correct risk calculation. In each setting under consideration, at least 11 out of 313 BCAC BC PRS loci, respectively 306 BRIDGES BC PRS loci, showed noticeably deviating AFs. These deviations were dependent on sequencing technology, variant caller, and calling mode and can be expected to affect the final BC risk calculations of the BOADICEA model implemented in CanRisk. Therefore, this study demonstrates the necessity to apply quality assurance not only in terms of sequencing coverage but also in terms of observed AFs in a sufficiently large cohort, when implementing PRSs in a routine diagnostic setting.

The presented results also point to potential solutions for improving genotyping performance with respect to the replication of expected AFs for several loci, these primarily include the use of alternative variant callers or consideration of proxy variants. The use of certain variant callers resulted consistently in noticeable deviating AFs, which were not observed for other callers. This concerned e.g., rs62485509 when using DRAGEN, and rs11268668 when using freebayes (Table 3). In each setting under investigation considering identical samples, the number of loci whose AFs match the expected AFs could be increased by variant-specific selection of the variant caller.

Comparison to large-scale population-specific data, such as gnomAD and 1000G High Coverage WGS, indicates that several PRS loci do not appear or appear with different alleles in NGS than in array-based genotyping. Here, four loci have been identified for which the use of alternative alleles could lead to the achievement of the intended, originally array-based determined AF, if NGS-based genotyping does not do so (Table 4). Two of these loci were absent in gnomAD v3.1.2 NFEs, which was also true for rs113778879 and rs572022984. As a potential workaround for rs113778879, which is an SNV, an overlapping 5 bp deletion was identified, but the observed AF exceeds the expected one by more than 0.1 (Supplementary Table 5). gnomAD SV v2.1 [24] reports a 1370 bp deletion starting at the same genomic position as rs572022984, namely DEL_2_27095, with an AF of 0.0417 in Europeans. However, genotyping of structural variants requires adapted variant calling approaches and therefore might be unfeasible within the scope of PRS genotyping in a routine diagnostic setting.

If no workarounds are available for loci showing noticeably deviating AFs, only imputation of the expected dosage according to CanRisk remains. This leads to smaller errors than omitting the locus from PRS calculation or setting the genotype to 0/0. However, each imputation causes a shift toward the mean PRS, and therefore imputations are applicable only up to a certain extent.

PRSs for calculating individual BC risks will continue to evolve. For example, currently, the Confluence Project^2^ aims to develop multi-ancestry PRSs. In addition, PRSs become also more and more relevant for the diagnostics of other diseases with a genetic component [25, 26]. The presented results underline that it would facilitate the implementation in clinical routine and thus also increase the reliability of genetic diagnostics if the design of future PRSs would be guided by the reproducibility of the expected AFs in addition to the observed effect sizes. A straightforward strategy to achieve this could be to ensure comparability of AFs in large-scale population databases, favorably based on different genotyping approaches, prior to including a locus in a PRS.

This study has limitations. Larger sample sizes may have resulted in more accurate estimators of AFs. Furthermore, there was a strong enrichment for samples derived from individuals with familial BC/OC, which may have resulted in deviating AFs due to genetic load rather than technical artifacts. The genetic background could explain, e.g., the aberrant (but concordant) AFs of rs55941023 in IHG data and of rs35054928 in CFBOC data. Despite checking family IDs, related individuals within a data set cannot be entirely excluded. Finally, no statement can be made about whether the described AF deviations would persist when using arrays for genotyping, since corresponding analyses are not (yet) performed in any of the GC-HBOC centers.

### Supplementary information

Supplementary Material

Supplementary Table 1

Suppplementary Table 2

Suppplementary Table 3

Suppplementary Table 4

Suppplementary Table 5

Suppplementary Table 6

## Data Availability

All data generated or analyzed during this study are included in this published article [and its Supplementary files].

## Footnotes

1 https://gnomad.broadinstitute.org.

2 https://confluence.cancer.gov.

## References

[1] Lakeman IM, Hilbers FS, Rodrìguez-Girondo M, Lee A, Vreeswijk MP, Hollestelle A, et al. Addition of a 161-SNP polygenic risk score to family history-based risk prediction: impact on clinical management in non-*BRCA1/2* breast cancer families. J Med Genet. 2019;56:581–9. 31186341 10.1136/jmedgenet-2019-106072

[2] Mavaddat N, Michailidou K, Dennis J, Lush M, Fachal L, Lee A, et al. Polygenic risk scores for prediction of breast cancer and breast cancer subtypes. Am J Hum Genet. 2019;104:21–34. 30554720 10.1016/j.ajhg.2018.11.002 PMC6323553

[3] Shieh Y, Hu D, Ma L, Huntsman S, Gard CC, Leung JW, et al. Breast cancer risk prediction using a clinical risk model and polygenic risk score. Breast Cancer Res Treat. 2016;159:513–25. 27565998 10.1007/s10549-016-3953-2 PMC5033764

[4] Borde J, Ernst C, Wappenschmidt B, Niederacher D, Weber-Lassalle K, Schmidt G, et al. Performance of breast cancer polygenic risk scores in 760 female *CHEK2* germline mutation carriers. J Natl Cancer Inst. 2021;113:893–9. 33372680 10.1093/jnci/djaa203 PMC8246885

[5] Borde J, Laitman Y, Blümcke B, Niederacher D, Weber-Lassalle K, Sutter C, et al. Polygenic risk scores indicate extreme ages at onset of breast cancer in female *BRCA1/2* pathogenic variant carriers. BMC Cancer. 2022;22:1–9. 35761208 10.1186/s12885-022-09780-1 PMC9238030

[6] Gallagher S, Hughes E, Wagner S, Tshiaba P, Rosenthal E, Roa BB, et al. Association of a polygenic risk score with breast cancer among women carriers of high-and moderate-risk breast cancer genes. JAMA Netw Open. 2020;3:e208501–e208501. 32609350 10.1001/jamanetworkopen.2020.8501 PMC7330720

[7] Kuchenbaecker KB, McGuffog L, Barrowdale L, Lee A, Soucy P, Healey S, et al. Evaluation of polygenic risk scores for breast and ovarian cancer risk prediction in *BRCA1* and *BRCA2* mutation carriers. J Natl Cancer Inst. 2017;109:djw302. 28376175 10.1093/jnci/djw302 PMC5408990

[8] Stiller S, Drukewitz S, Lehmann K, Hentschel J, Strehlow V. Clinical impact of polygenic risk score for breast cancer risk prediction in 382 individuals with hereditary breast and ovarian cancer syndrome. Cancers. 2023;15:3938. 37568754 10.3390/cancers15153938 PMC10417109

[9] Carver T, Hartley S, Lee A, Cunningham AP, Archer S, Babb de Villiers C, et al. CanRisk tool – a web interface for the prediction of breast and ovarian cancer risk and the likelihood of carrying genetic pathogenic variants. Cancer Epidemiol Biomark Prev. 2021;30:469–73. 10.1158/1055-9965.EPI-20-1319 PMC7611188 33335023

[10] Lee A, Mavaddat N, Wilcox AN, Cunningham AP, Carver T, Hartley S, et al. BOADICEA: a comprehensive breast cancer risk prediction model incorporating genetic and nongenetic risk factors. Genet Med. 2019;21:1708–18. 30643217 10.1038/s41436-018-0406-9 PMC6687499

[11] Tüchler A, De Pauw A, Ernst C, Anota A, Lakeman IMM, Dick J, et al. Clinical implications of incorporating genetic and non-genetic risk factors in CanRisk-based breast cancer risk prediction. Breast 2024;73:103615. 38061307 10.1016/j.breast.2023.103615 PMC10749276

[12] Carver T. CanRisk knowledgebase. 2022. https://canrisk.atlassian.net/wiki/spaces/FAQS/pages/35979266/What+variants+are+used+in+the+PRS. Accessed 30 Nov 2022.

[13] Mavaddat N, Ficorella L, Carver T, Lee A, Cunningham AP, Lush M, et al. Incorporating alternative polygenic risk scores into the BOADICEA breast cancer risk prediction model. Cancer Epidemiol Biomark Prev. 2023;32:422–7. 10.1158/1055-9965.EPI-22-0756 PMC9986688 36649146

[14] Kiialainen A, Karlberg O, Ahlford A, Sigurdsson S, Lindblad-Toh K, Syvänen AC. Performance of microarray and liquid based capture methods for target enrichment for massively parallel sequencing and SNP discovery. PLoS ONE. 2011;6:e16486. 21347407 10.1371/journal.pone.0016486 PMC3036585

[15] Sulonen AM, Ellonen P, Almusa H, Lepistö M, Eldfors S, Hannula S, et al. Comparison of solution-based exome capture methods for next generation sequencing. Genome Biol. 2011;12:1–18. 10.1186/gb-2011-12-9-r94 PMC3308057 21955854

[16] Teer JK, Bonnycastle LL, Chines PS, Hansen NF, Aoyama N, Swift AJ, et al. Systematic comparison of three genomic enrichment methods for massively parallel DNA sequencing. Genome Res. 2010;20:1420–31. 20810667 10.1101/gr.106716.110 PMC2945191

[17] Yi M, Zhao Y, Jia L, He M, Kebebew E, Stephens RM. Performance comparison of SNP detection tools with Illumina exome sequencing data – an assessment using both family pedigree information and sample-matched SNP array data. Nucleic Acids Res. 2014;42:e101–e101. 24831545 10.1093/nar/gku392 PMC4081058

[18] Li H. Toward better understanding of artifacts in variant calling from high-coverage samples. Bioinformatics. 2014;30:2843–51. 24974202 10.1093/bioinformatics/btu356 PMC4271055

[19] Reis AL, Deveson IW, Madala BS, Wong T, Barker C, Xu J, et al. Using synthetic chromosome controls to evaluate the sequencing of difficult regions within the human genome. Genome Biol. 2022;23:1–24. 35022065 10.1186/s13059-021-02579-6 PMC8753822

[20] Stoler N, Nekrutenko A. Sequencing error profiles of Illumina sequencing instruments. NAR Genom Bioinform. 2021;3:lqab019. 33817639 10.1093/nargab/lqab019 PMC8002175

[21] Gudmundsson S, Singer-Berk M, Watts NA, Phu W, Goodrich JK, Solomonson M, et al. Variant interpretation using population databases: lessons from gnomAD. Hum Mutat. 2022;43:1012–30. 34859531 10.1002/humu.24309 PMC9160216

[22] Karczewski KJ, Francioli LC, Tiao G, Cummings BB, Alföldi J, Wang Q, et al. The mutational constraint spectrum quantified from variation in 141,456 humans. Nature. 2020;581:434–43. 32461654 10.1038/s41586-020-2308-7 PMC7334197

[23] Sherry ST, Ward MH, Kholodov M, Baker J, Phan L, Smigielski EM, et al. dbSNP: the NCBI database of genetic variation. Nucleic Acids Res. 2001;29:308–11. 11125122 10.1093/nar/29.1.308 PMC29783

[24] Collins RL, Brand H, Karczewski KJ, Zhao X, Alföldi J, Francioli LC, et al. A structural variation reference for medical and population genetics. Nature. 2020;581:444–51. 32461652 10.1038/s41586-020-2287-8 PMC7334194

[25] Adeyemo A, Balaconis MK, Darnes DR, Fatumo S, Moreno PG, Hodonsky CJ, et al. Responsible use of polygenic risk scores in the clinic: potential benefits, risks and gaps. Nat Med. 2021;27:1876–84. 34782789 10.1038/s41591-021-01549-6

[26] Sugrue LP, Desikan RS. What are polygenic scores and why are they important? JAMA. 2019;321:1820–1. 30958510 10.1001/jama.2019.3893

